# Supplementary material for: The relationship between family environments growing up and behavioral health among LGBTQ+ adults: The mediating role of internalized homonegativity
Source: PLoS One. 2025 Jun 27;20(6):e0327370. doi: 10.1371/journal.pone.0327370 (PMC12204554; doi:10.1371/journal.pone.0327370)
Supplement: Attachment — Submitted filename: Table for Responses to Reviewers.docx [file pone.0327370.s003.docx]

**Table for Responses to Reviewers**

| **Reviewers’ Comment** | **Authors’ Response** | **Location in Manuscript** |
| --- | --- | --- |
| **Editor** | | |
| Please provide additional details regarding participant consent. In the ethics statement in the Methods and online submission information, please ensure that you have specified (a) whether consent was informed and (b) what type you obtained (for instance, written or verbal, and if verbal, how it was documented and witnessed). If your study included minors, state whether you obtained consent from parents or guardians. If the need for consent was waived by the ethics committee, please include this information. | Thank you for the reminder. We updated the consent process in the Method section and included the IRB number. | Line 197-199, 202-203 |
| Please provide an amended statement that declares *all* the funding or sources of support (whether external or internal to your organization) received during this study, as detailed online in our guide for authors at http://journals.plos.org/plosone/s/submit-now.  Please also include the statement “There was no additional external funding received for this study.” in your updated Funding Statement.  Please include your amended Funding Statement within your cover letter. We will change the online submission form on your behalf. | We would like to revise our funding statement to: *Research reported in this article was supported by the National Institute of Minority Health and Health Disparities of the National Institutes of Health (https://www.nimhd.nih.gov/) under award number R21MD012687. W.H. is the author who received this award. The funders had no role in study design, data collection and analysis, decision to publish, or preparation of the manuscript. There was no additional external funding received for this study.* | N/A |
| Please remove any funding-related text from the manuscript and let us know how you would like to update your Funding Statement. | We have deleted the funding information from the manuscript. | N/A |
| In the online submission form, you indicated that your data is available only on request from a third party. Please note that your Data Availability Statement is currently missing the name of the third party contact or institution and contact details for the third party, such as an email address or a link to where data requests can be made. Please update your statement with the missing information. | We have revised the statement in the submission system: *The data may be made available upon request by contacting the Principal Investigator, W.H. (wjhall@email.unc.edu).* | Online submission |
| I found that was not addressed is the potential intersectional influence of race, ethnicity, and immigrant background on the mediating role of internalized homonegativity and behavioral health outcomes. Your sample presents significant demographic diversity and includes these variables in regression models, but further interpretation of how these intersecting identities may compound or mitigate the impact of familial homophobia and internalized stigma could provide valuable insights. If this is beyond the scope of your research, this should be discussed clearly as to why. | Thank you for suggesting the important concept of intersectionality in relation to the diversity of our sample. While analyses of interactions between demographic variables and IH are important for LGBTQ+ and family research, they are beyond the scope of this paper. We agree that this topic warrants further exploration in future studies and have added this point to the discussion section. | Line 468-472 |
| **Reviewer #1** | | |
| Please ensure that page numbers and/or line numbers are correctly indicated. | Thank you for the reminder. Page numbers and line numbers are added. | N/A |
| Be more precise when you used “world” in the sentence “Even though societies adopt more policies protecting LGBTQ+ rights and discussing LGBTQ+ issues openly more than before, heteronormativity and cisnormativity remain dominant in the world and affect social systems and LGBTQ+ people’s health and development (Mills-Koonce et al., 2018; Van Bergen et al., 2021). Maybe you talk about countries. It could be interesting to add that some of them criminalize LGBTQ+ people. Please detail in which ways it impact heath and development of LGBTQ+ people | Thank you for your suggestion. We have added some literature about the details and replaced the word “world” to “countries worldwide” | Line 53-59 |
| Add reference for: “IH is also considered a consequence of heteronormative societal beliefs and affects one’s behavioral Health” | Thank you for the reminder. We have added the citations. | Line 67-68 |
| In the Family Environments for LGBTQ+ section • Maybe you could add a subsection title as “Social environment” for first paragraph as a broader picture or modify the section by “Social and Family Environments for LGBTQ+” • “Despite important findings about influences of family and caregiver interactions with LGBTQ+ children’s behavioral health outcomes, existing studies largely focus on individual-level factors (e.g., caregiver factors).” Please add examples of individual level factors and references | Thank you for the suggestion. We have changed the section title to “Social and Family Environments for LGBTQ+” and added literature about family individual level factors. | Line 86, 105-108 |
| Please define precisely which scores are calculated for each scale. For example, you add final scores ranged from 5 to 25 for Homophobic messages received from family. Add for others scales and add these informations in table 1 for each scale result to understand your results. Range for scales in table 1 | Thank you for pointing out the lack of information in the manuscript. Average scores were used for all the scales. We have modified the texts and added the minimum and maximum scores in Table 1. | Line 213-214, 220, 227, 234-235, 244, 304 |
| Openly LGBTQ+ people in family or not: • How do you define family to answer this question: When you were growing up, how many openly LGBTQ+ people were there in your family?” • Response options were 0, 1 or 2, 3 or 4, or 5 or more. Given the response distribution, this variable was recoded 0 (0) and 1 (1 or more). Give example for this variable recoding. By the way, it is not defined in results section. | Thank you for raising this important point regarding the definition of family. The researchers who designed the survey did not provide a fixed definition of family in the survey. Therefore, the question relies on participants’ understanding of family; however, use of “when you were growing up” implies that the question refers to the family of origin. Nonetheless, we have added this point to the limitation section.  Regarding the coding of this variable, we modified how the response options were labeled in the main text to make it clearer. Given the response distribution, this variable was recoded 0 (No openly LGBTQ+ people in family) and 1 (Having openly LGBTQ+ people in family). | Line 255-256, 445-449, 466-468 |
| Data analysis: did you think of a correction for multiple models applied to data? | While we did not apply formal corrections for multiple models, our analytic models were guided by Hatzenbuehler’s (2009) *Psychological Mediation Framework* and prior empirical findings as discussed in the introduction section, which helped limit the number of tests and reduce the risk of data-driven results. We acknowledge this as consideration and have noted it as a limitation in the discussion section. | Line 451-455, 477-479 |
| Pages 13-14: please put table in one page | We have adjusted the table. | Line 317 |
| You discussed results about having openly LGBTQ+ family member in page 17 but don’t mentioned it in limitations. Please discussed this definition of family and variable recoding | Thank you for the important reminder. As noted above, lack of a standardized definition of “family” may leave some openness to interpretation. We acknowledge this as a limitation and have noted it in the discussion section. | Line 255-256, 445-449, 466-468 |
| Please discussed study context of covid and potential implications of this specific period in your study. For example responses of participants if they coming back to their family and locked with them in that period. | Thank you for this important comment. Data for this study were collected during the COVID-19 pandemic, a period that may have shaped participants’ experiences of family dynamics and mental health. We acknowledge that the unique circumstances of the pandemic may limit the generalizability of our fundings to non-pandemic periods. We have added this as a limitation in the discussion section, and noted this specific period in the implication section. | Line 426-430, 437-442 |
| **Reviewer #2** | | |
| While the review of the research literature covers many relevant studies, there needs to be some reference to the “minority stress” and homophobic “micro-aggression” research literatures which consolidate many of these findings around a few central concepts. | Thank you for the suggestion. We did utilize concepts of minority stress and micro-aggressions in the study but did not explicitly indicate them. We have added related literature to the manuscript. | Line 44-47, 98-100 |
| While the study reports demographic rates for cis-gender/trans and sexual identities, there is curiously no mention of adult gender identity distinguishing men and women. Was gender used in the analysis? Were there any gender differences in either the familial or mental health variables? Related to this, gay and lesbian cannot simply be treated as a single unitary identity. Were there any differences between gay and lesbian categories? Also, what is the gender breakdown of bi/pansexual respondents? Given that the majority of the sample identified as bi/pansexual and had significantly higher rates of anxiety and depression, did gay, lesbian, or trans people present different profiles regarding IH or indicators of familial homophobia? | Thank you for raising this important point regarding demographic differences. Unfortunately, the gender identity variable was coded cisgender/transgender and was not broken down by cisgender-men, cisgender-women, etc. We agree that more specific disaggregation by gender identity (as well as other identities [e.g., bi/pansexual]) would provide potentially interesting subgroup comparisons. However, the original coding of the data by the survey researchers limits our analysis. We have noted this as limitation and future direction. | Line 455-457, 462-463, 468-472 |
| Given that increasing age was associated with lower anxiety/depression (in accord with other studies) and age can be associated with sexual identity, particularly bisexual identity, was age controlled for in reporting rates of anxiety or depression? In other words, how much of these bisexual rates are accounted for by age? | All the models in the study controlled for age, racial/ethnic identity, gender identity, sexual orientation, immigrant background, and geographic area. However, the interactions between demographics (e.g., age X sexual identity) are beyond the scope of the paper. We agree that this topic warrants further exploration in future studies and have added this point to the discussion section. | Line 468-472 |
| In the Implications section, which gestures in the direction of “inclusive practices in therapy, support programs, and educational initiatives [that] would help mitigate the negative impacts of childhood environmental homophobia,” it should be noted that there is significant research on the supportive effects of Gay/Straight Alliances (or equivalent) and anti-bullying programs in schools in improving the lives of LGBTQ+ youth. | Thank you for pointing out the importance of implications for action. We have added these suggested examples to the manuscript. | Line 412-420 |
| Editorial notes: • intro: should be “Trevor Project” • family environment section: should be “health outcomes in adulthood have been under-researched” • p 15 should be “LGBTQ+ people in one’s family did” | Thank you for editing. The “Trevor Project” in the intro was in an in-text citation, and it was removed after the manuscript was transferred into Vancouver citation format. The other two typos have been corrected. | Line 110, 334 |
